# Years of life lost to incarceration: inequities between Aboriginal and non-Aboriginal Canadians

**DOI:** 10.1186/1471-2458-14-585

**Published:** 2014-06-11

**Authors:** Akwasi Owusu-Bempah, Steve Kanters, Eric Druyts, Kabirraaj Toor, Katherine A Muldoon, John W Farquhar, Edward J Mills

**Affiliations:** 1 Centre of Criminology and Sociolegal Studies, University of Toronto, 14 Queen’s Park Crescent West, Toronto M5S 3 K9 Ontario, Canada; 2 School of Population and Public Health, The University of British Columbia, 2206 East Mall, Vancouver, British Columbia V6T 1Z3, Canada; 3 Faculty of Health Sciences, University of Ottawa, 25 University Private, Ottawa, Ontario K1N 6 N5, Canada; 4 Stanford Prevention Research Center, Stanford University, 291 Campus Drive, Stanford, California 94305-5101, USA

**Keywords:** Prison, Incarceration, Aboriginal, Public health

## Abstract

**Background:**

Aboriginal representation in Canadian correctional institutions has increased rapidly over the past decade. We calculated “years of life lost to incarceration” for Aboriginal and non-Aboriginal Canadians.

**Methods:**

Incarceration data from provincial databases were used conjointly with demographic data to estimate rates of incarceration and years of life lost to provincial incarceration in (BC) and federal incarceration, by Aboriginal status. We used the Sullivan method to estimate the years of life lost to incarceration.

**Results:**

Aboriginal males can expect to spend approximately 3.6 months in federal prison and within BC spend an average of 3.2 months in custody in the provincial penal system. Aboriginal Canadians on average spend more time in custody than their non-Aboriginal counterparts. The ratio of the Aboriginal incarceration rate to the non-Aboriginal incarceration rate ranged from a low of 4.28 in Newfoundland and Labrador to a high of 25.93 in Saskatchewan. Rates of incarceration at the provincial level were highest among Aboriginals in Manitoba with an estimated rate of 1377.6 individuals in prison per 100,000 population (95% confidence interval [CI]: 1311.8 – 1443.4).

**Conclusions:**

The results indicate substantial differences in life years lost to incarceration for Aboriginal versus non-Aboriginal Canadians. In light of on-going prison expansion in Canada, future research and policy attention should be paid to the public health consequences of incarceration, particularly among Aboriginal Canadians.

## Background

Canada has a relatively low rate of incarceration compared to its American neighbours. It ranks in the middle of the Organization for Economic Cooperation and Development (OECD) countries with an incarceration rate of 117 prisoners per 100,000 population [1]. The size of Canada’s federal prison population has increased modestly in the past decade. This increase, however, has not been uniform across racial groups. The Canadian Aboriginal prison population has risen by almost 40%, while the non-Aboriginal prison population has risen by just over 2% [2]. For Aboriginal women the increase over this time period was 85%, while the Aboriginal male prison population increased by 26% [2].

Previous American research has examined the impact of incarceration by race on life years [3]. Importantly, this research identifies substantial racial differences in “years of life lost to incarceration” in the United States. The objective of the present analysis is to use similar methods in a Canadian context by focusing on differences in years of life lost to incarceration for Aboriginal and non-Aboriginal peoples, where “years of life lost to incarceration” is defined as the years spent incarcerated. This line of inquiry is more challenging in Canada with limited access to correctional and demographic data that is disaggregated by race.

## Methods

We first compared rates of incarceration between Aboriginal and non-Aboriginal Canadians for both the provincial and federal correctional systems in Canada. Second, we calculated the years of life lost to incarceration for these two population groups for the provincial prison system in British Columbia (BC), and the Canada-wide federal correctional system. Years of life lost to incarceration represents the number of years spent incarcerated and therefore lost to incarceration.

### Data sources

Canada has a two-tiered correctional system. Offenders sentenced to two or more years are sent to the federal system, and offenders sentenced to less than two years or who are detained before trial are sent to the provincial system. Disaggregated incarceration data by Aboriginal status are not readily available (except for the BC provincial system), and so, we requested these data for the federal system and all other provincial systems through the federal Access to Information Act and provincial Freedom of Information Acts [4]. These data make use of the *Employment Equity Act* (1995) definition of Aboriginal status, which is inclusive of Métis [5].

Table 1 provides a summary of all data elements used in the analyses, their sources, dates of retrieval, and their analytical use. Incarceration data were provided to us as admissions (the total number of persons admitted to custody in a given year) and/or one-day counts (the total number of persons in custody on an exact date). The type of data we received was at the discretion of the data provider. One-day counts were available for Newfoundland and Labrador, Ontario, Manitoba, Saskatchewan, Alberta, the Yukon and Nunavut. (Note that data provided by Prince Edward Island, Nova Scotia, New Brunswick, Quebec, and Northwest Territories did not include one-day counts, and were therefore excluded from the present analyses.) One-day count data for BC were obtained directly from the BC Justice publicly available database [6].

**Table 1:** Components and data sources for incarceration rate and years of life lost to incarceration analyses

**Component**	**Analytical use**	**Source**
**Institutional Data**		
Newfoundland one-day counts	Estimate rates of incarceration	Newfoundland and Labrador Department of Justice; Received September 26, 2011
Prince Edward Island	No data available	Prince Edward Island Department of Justice and Public Safety; Received September 12, 2011
Nova Scotia annual admission counts	Data provided by this province was not compatible with analytic methods	Nova Scotia Department of Justice; September 15, 2011
New Brunswick annual admission counts	Data provided by this province was not compatible with analytic methods	New Brunswick Department of Public Safety Community and Correctional services; Received September 13, 2011
Quebec annual admission counts	Data provided by this province was not compatible with analytic methods	Ministre de la Securite Publique Quebec; September 8, 2011
Ontario one-day counts	Estimate rates of incarceration	Ministry of Community Safety and Correctional Services; Received December 14, 2011
Manitoba one-day counts	Estimate rates of incarceration	Manitoba Justice; Received September 19, 2011
Saskatchewan one-day counts	Estimate rates of incarceration	Ministry of Corrections, Public Safety and Policing; Received on August 24, 2011
Alberta one-day counts	Estimate rates of incarceration	Alberta Solicitor General and Public Security; Received on September 13, 2011
British Columbia one-day counts	Estimate YLLP (N_x_P_ix_) and rates of incarceration	JusticeBC Dashboards – Corrections Adults Custody. Retrieved for April 1, 2011
Yukon one-day counts	Estimate rates of incarceration	Department of Justice; Received on September 8, 2011
Nunavut one-day counts	Estimate rates of incarceration	Department of Justice; Received on September 19, 2011
Northwest Territories annual admission counts	Data provided by this province was not compatible with analytic methods	Northwest Territories Justice; Received September 16, 2011
Canada one-day counts	Estimate YLLP (N_x_P_ix_) and rates of incarceration	Correctional Service Canada; Received on October 25, 2011
Sex distribution among federally imprisoned Aboriginals and non-Aboriginals	Estimate YLLP (N_x_P_ix_)	Public Safety Canada. 2012 Annual Report: Corrections and Conditional Release, Statistical Overview. 2011 results used.
Age distribution among federally imprisoned Aboriginals and non-Aboriginals	Estimate YLLP (N_x_P_ix_)	Public Safety Canada. 2012 Annual Report: Corrections and Conditional Release, Statistical Overview. 2011 results used.
**Demographic Data**		
2007-2009 Life tables for total Canadian population disaggregated by province and sex	To obtain l_x_ in using the Sullivan method to estimate YLLP	Statistics Canada. Annual Demographic Estimates: Canada, Provinces and Territories. Catalogue no. 91-215-X
Probability of survival by age and sex for aboriginals	To obtain l_x_ in using the Sullivan method to estimate YLLP	Statistic Canada: Table 109–5402 Probability of survival at various ages, by population group and sex, Canada
Demographics by age, sex and Aboriginal status	To obtain N_x_ in using the Sullivan method to estimate YLLP and to estimate rates	Statistics Canada. 2011 National Household Survey. Catalogue Number: 99-011-X2011027

Note: 1) YLLP, years of life lost to prison; 2) N_x_P_ix_, number of individuals with disability (incarceration); 3) N_x_, number of persons in population; 4) l_x_, probability of surviving to the age at beginning of the age category; 5) No data was provided by Prince Edward Island 6) Data provided by Nova Scotia, New Brunswick, Quebec, and Northwest Territories did not include one-day counts, and were therefore excluded from the present analyses.

Although one-day count data for the federal system were obtained from the Correctional Service of Canada (CSC) through the Access to Information Act, these data were not disaggregated by sex and age. We therefore made use of additional data from the Public Safety Canada’s 2012 annual report [7] to construct age- and sex-specific distributions by Aboriginal status.

Additional demographic data needed to perform our analysis were acquired through Statistics Canada. Aboriginal and non-Aboriginal population sizes for each province were obtained from the 2011 National Household Survey and used as the population denominator [8]. Probability of survival to various ages according to race and sex were obtained from the 2007–2009 life tables and CANSIM (Canadian socio-economic information management system) table 109–5402 [9,10].

### Data analyses

The available one-day count data were used conjointly with the population estimates to calculate rates of incarceration by Aboriginal status for the provincial and federal systems. These rates of incarceration were calculated for the entire adult population aged 18 years and older. Rates of incarceration were calculated by dividing the one-day counts by the adult population, and then multiplying by 100,000. The corresponding 95% confidence intervals (CI) were constructed using z-intervals for proportions.

The one-day counts, population estimates, and probability of survival data were used to calculate the years of life lost to incarceration using the Sullivan method [11]. The Sullivan method lends itself well to situations such as this one, where mortality and a specific condition (in our case, incarceration) are collected separately. The years of life lost to incarceration calculations were only completed for the BC system and the federal system because age- and sex-specific distributions of incarceration were obtainable for these datasets.

An abridged Sullivan’s life table was used with 5-year age categories. The only exception was a 2-year category for ages 18–19 years. For each age category, the age-specific incarceration prevalence was used to adjust the person-years of the age category – obtained using the length of each age category, the number of people in the age group, and the probability of survival prior to the age category of interest. In this fashion, the total number of years spent in custody and the total number of years lived out of custody were separated within the total number of life years. Summing these across age groups for each sex and each race provided prison-free life expectancies, imprisoned life expectancies and overall life expectancies. Our calculations were restricted to those aged 18–55 years in BC and 18–65 years in Canada. This difference in age spans between the BC provincial analysis and the Canada-wide federal analysis is due data availability. Rate ratios (RR) were used to compare Aboriginal to non-Aboriginal Canadians.

## Results

Table 2 presents rates of incarceration for those aged 18 years and older in 2011 by Aboriginal status for provincial prison systems in Newfoundland/Labrador, Ontario, Manitoba, Saskatchewan, Alberta, BC, Yukon and Nunavut, and for the Canadian federal correctional system. The provincial incarceration RR comparing Aboriginal to non-Aboriginal Canadians varied from 4.28 per 100,000 population in Newfoundland and Labrador to 25.93 per 100,000 population in Saskatchewan. Rates of incarceration in the provincial systems were highest among Aboriginals in Manitoba, with an estimated rate of 1,377.6 individuals in custody per 100,000 population (95% CI: 1,311.8 – 1,443.4). Non-Aboriginals in the Newfoundland provincial system had the lowest overall rate (47.3 [95% CI: 41.9 – 52.6] persons per 100,000 population). The provincial prison system in the territory of Nunavut had a lower estimated rate than Newfoundland, but is based on a sample size of 1. Rates of incarceration among Aboriginals in the Manitoba, Saskatchewan, Alberta and Yukon provincial systems were all above 800 persons in custody per 100,000 population.

**Table 2:** Rates of incarceration (per 100,000 population) for adults aged 18 years or older

	**Aboriginal**		**Non-Aboriginal**		**Rate ratio (95% CI)**
	**Rate of incarceration (95% CI)**	**Total adult population**	**Rate of incarceration (95% CI)**	**Total adult population**	
**Provincial penal systems**					
Newfoundland/Labrador	202.2 (147.3-257.1)	25713	47.3 (41.9-52.6)	637,064	4.28 (4.09-4.48)
Prince Edward Island	NA	1,484	NA	106,952	N/A
Nova Scotia	NA	23,735	NA	710,122	NA
New Brunswick	NA	15,588	NA	579,480	NA
Quebec	NA	101,096	NA	6,080,120	NA
Ontario	468.1 (438.9-497.3)	210,426	75.4 (73.7-77.2)	9,749,071	6.23 (6.22-6.25)
Manitoba	1377.6 (1311.8-1443.4)	120,645	75.1 (69.0-81.2)	775,341	18.59 (18.50-18.67)
Saskatchewan	1260.1 (1188.7-1331.6)	93,643	49.2 (43.9-54.5)	676,566	25.93 (25.73-26.13)
Alberta	800.3 (753.7-846.9)	140,320	67.7 (64.5-70.9)	2,607,172	11.91 (11.87-11.94)
British Columbia	305.1 (277.9-332.3)	157,669	67.3 (64.5-70.0)	3,325,281	4.54 (4.52-4.57)
Yukon	1285.6 (984.4-1587.3)	5,366	67.3(32.1-102.6)	20,789	19.34 (15.80-23.67)
Nunavut	639.5 (514.5-764.4)	15,638	NA*	3,793	24.37 (N/A)
Northwest Territories	NA	13,831	NA	15,945	NA
**Canada federal penal system**					
Canada	330.4 (318.7 342.1)	925,146	44.6 (43.8 45.4)	25,038,216	7.43 (7.42-7.44)

Note: CI, confidence interval; NA: not applicable due to unavailable data.

*Only one incarceration reported.

Table 3 shows life years lost to incarceration by Aboriginal status and sex for the provincial system in BC and within the federal correctional system throughout Canada. Non-Aboriginal females spent the least amount of time in custody with an average of 0.005 years (1.8 days) in BC prisons and 0.002 years (0.7 days) in federal penitentiaries. On average, Aboriginal males spent 3.75 times longer in provincial custody than non-Aboriginal males in BC and 6.18 times longer in federal custody. Aboriginal males spend an average of 3.6 months incarcerated in the federal system and within BC spend an average of 3.2 months incarcerated in the provincial prison system. Aboriginal females spend on average 6.4 times longer incarcerated in the provincial prison system within BC and 9 times longer in the federal system across Canada than non-Aboriginal females. Aboriginal males can expect to spend 3.8 and 6.2 times longer in custody than non-Aboriginal males within the provincial and federal systems, respectively.

**Table 3:** Years of life lost to incarceration

	**Life expectancy (in years)**	**Non-prison component (in years)**	**Prison component (in years)**
**British Columbia provincial penal system**			
Male			
Aboriginal	72.03	71.76	0.270
Non-Aboriginal	79.80	79.73	0.072
Female			
Aboriginal	76.30	76.27	0.032
Non-Aboriginal	84.03	84.03	0.005
**Canada federal penal system**			
Male			
Aboriginal	72.03	71.72	0.303
Non-Aboriginal	79.80	79.75	0.049
Male			
Aboriginal	76.30	76.28	0.018
Non-Aboriginal	83.42	83.42	0.002

Note: 1) All standard errors below 0.013; 2) Calculations were restricted to those aged 18–55 years in BC and 18–65 years in Canada. This difference in age spans between the BC provincial analysis and the Canada-wide federal analysis is due data availability.

## Discussion and conclusions

This analysis indicates that an Aboriginal Canadian can expect to lose considerably more life-years to incarceration than a non-Aboriginal Canadian. Our analysis further indicates that Aboriginals are over-represented as inmates in federal and provincial correctional institutions. At the federal level, Aboriginals lose six times as many life years to incarceration than non-Aboriginals. Within the province of BC, Aboriginal Canadians lose approximately four times as many life years to incarceration as non-Aboriginal-Canadians. These relative differences are more pronounced in women than men, but the absolute differences remain larger among men.

There is a growing body of evidence establishing the serious negative health consequences associated with incarceration [12-14]. The health risks associated with incarceration include physical and sexual violence, sex without condoms, and the sharing of needles, contributing to the transmission of communicable diseases [15-17]. Signaled clearly by the recent assent of the *Safe Streets and Communities Act (Bill C-10),* which among other provisions, increases mandatory minimum sentences for marijuana offences, the Canadian Government is introducing harsher laws and increasing the use of incarceration in Canada. We believe that it is critical to consider the serious health consequences associated with the use of incarceration as a means of punishment. This is particularly true for Aboriginal Canadians, a group already provided special consideration in criminal justice matters as a result of their historical disadvantage. Population estimates predict that the Aboriginal population aged 20-29 in Canada will grow by 40% by 2017 [18]. This is troubling because this age group contains the greatest proportion of Aboriginal offenders; Aboriginals therefore stand to be disproportionately impacted by Bill C-10 [19].

Aside from the quantifiable losses associated with incarceration there are a variety of societal impacts. These include, but are not limited to, increased aggression and behavioral problems in the children of those incarcerated, effects on the family structure, limits on future economic opportunities, and reduced community safety [20-24]. From this we can infer that the disproportionate levels of Aboriginal incarceration do not only have adverse health consequences, but many negative social consequences as well.

Aboriginal over-representation in the correctional population is highest in provincial institutions in Manitoba and Saskatchewan at rates of 1,377.6 and 1,260.1 per 100,000 population, respectively. However, due to limited data, our analysis could only focus on the province of BC, where Aboriginals are incarcerated at a rate of 305.1 per 100,000 population. Therefore, this provides a conservative estimate of life-years lost to provincial incarceration amongst Aboriginal Canadians. The Sullivan method was used because it is the most applicable method when using prevalence data. However, if more extensive data were available, better methods, that reflect transitions in and out of prison, could be used to estimate life years lost to incarceration with more precision and less bias. The federal data, contrary to the BC provincial data, did not differentiate the age distribution between sexes within each racial group. This may introduce minimal bias as males make up 96% of federal inmates. Finally, the prevalence was estimated using one-day counts that may differ throughout the year. This extra source of variability is captured in the construction of the confidence intervals, but should be interpreted with some degree of caution.

This study presents a unique approach to examining Aboriginal people’s experiences with incarceration in Canada. Previous research has focused on Aboriginal representation within Canadian prison systems as well as rates of incarceration compared to other racial groups [25,26]. To the best of our knowledge, this is the first study to estimate the impact of over-incarceration among Aboriginal Canadians throughout an individual’s lifetime. Furthermore, by framing Aboriginal over-incarceration as a public health issue this analysis aims to bring new empirical evidence and renewed attention to a persisting Canadian problem.

The Canadian Federal Correctional Investigator recently criticized the Correctional Service of Canada for making little progress in closing the gaps in adverse prison outcomes between Aboriginal and non-Aboriginal offenders [27]. There is a clear need to examine the over-representation of Aboriginals at all stages of criminal justice processing, from the police, through the courts to the correctional systems. However, as this analysis clearly illustrated, there is a critical need for better, more extensive data on the matter to inform evidence-based policy.

## Competing interests

The authors declare that they have no competing interests.

## Authors’ contributions

All authors took part in the study design, the analysis, and the interpretation of the data. Data were acquired by AO-B. The first draft of the manuscript was written by AO-B, and was critically revised for important intellectual content by all authors. The study guarantor is EM. All authors approved the final manuscript for publication.

## Pre-publication history

The pre-publication history for this paper can be accessed here:

http://www.biomedcentral.com/1471-2458/14/585/prepub
